# Ship Spatiotemporal Key Feature Point Online Extraction Based on AIS Multi-Sensor Data Using an Improved Sliding Window Algorithm

**DOI:** 10.3390/s19122706

**Published:** 2019-06-16

**Authors:** Miao Gao, Guo-You Shi

**Affiliations:** 1 Navigation College, Dalian Maritime University, Dalian 116026, China; gaomiao4566@foxmail.com; 2 Collaborative Innovation Research Institute of Autonomous Ship, Dalian Maritime University, Dalian 116026, China; 3 Key Laboratory of Navigation Safety Guarantee of Liaoning Province, Dalian 116026, China

**Keywords:** big data mining, spatiotemporal, ship behavior, key feature point, feature online extraction, AIS, sliding window algorithm

## Abstract

Large volumes of automatic identification system (AIS) data provide new ideas and methods for ship data mining and navigation behavior pattern analysis. However, large volumes of big data have low unit values, resulting in the need for large-scale computing, storage, and display. Learning efficiency is low and learning direction is blind and untargeted. Therefore, key feature point (KFP) extraction from the ship trajectory plays an important role in fields such as ship navigation behavior analysis and big data mining. In this paper, we propose a ship spatiotemporal KFP online extraction algorithm that is applied to AIS trajectory data. The sliding window algorithm is modified for application to ship navigation angle deviation, position deviation, and the spatiotemporal characteristics of AIS data. Next, in order to facilitate the subsequent use of the algorithm, a recommended threshold range for the corresponding two parameters is discussed. Finally, the performance of the proposed method is compared with that of the Douglas–Peucker (DP) algorithm to assess its feature extraction accuracy and operational efficiency. The results show that the proposed improved sliding window algorithm can be applied to rapidly and easily extract the KFPs from AIS trajectory data. This ability provides significant benefits for ship traffic flow and navigational behavior learning.

## 1. Introduction

**Figure 1:**
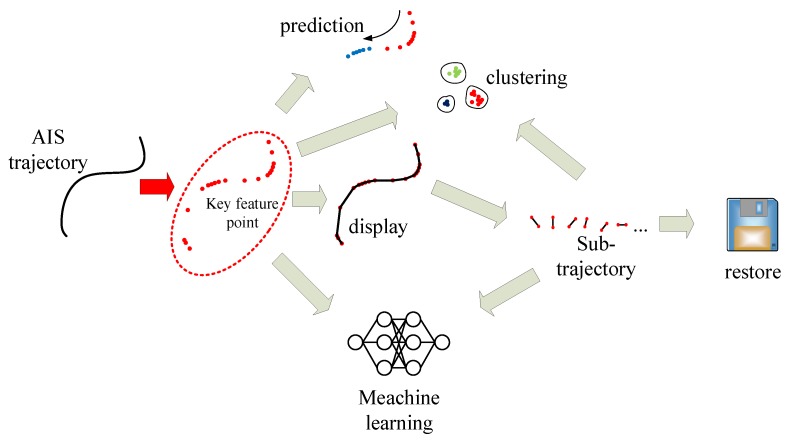
Multiple applications of key feature points (KFPs).

According to the SOLAS (International Convention for Safety of Life at Sea) convention, since 2002, an increasing number of ships have been required to install automatic identification system (AIS) equipment onboard [1]. The basic framework of coastal AIS base stations has been developed over almost 10 years of constructing special frameworks for land-based stations worldwide. A large volume of AIS trajectory data has been accumulated owing to the increasing number of ship terminals, the high frequency of ship terminal information forecasts [2], and improved data collection. A large volume of data provides new ideas and methods for the effective application of AIS trajectory data for improving the safety and efficiency of maritime traffic [3,4], which has become an active research topic with the development of big data analysis techniques. However, the volume, velocity, variety, value, and veracity (5V) characteristics of big data are a double-edged sword; although big data can provide several new research directions and ideas, research on hardware devices and algorithms is a significant challenge. The massive volumes cause great difficulties in all aspects of data storage, transmission, analysis, calculation, display, etc. The low value of individuals causes the learning efficiency to decrease and the relevance to become poor. The variety of data and multi-dimensionality have higher requirements for algorithm complex. The high-velocity collection and data growth have higher requirements for algorithm processing efficiency. Therefore, the ship trajectory key feature point (KFP) online extraction from trajectory data plays an extremely important role in the study of the navigational behavior of ships. As shown in Figure 1, most of the research based on AIS big data is essentially conducted by means of KFPs, such as behavior prediction [5,6], clustering [7], data restoration [8], display [9], data mining [10], and machine learning theory [11]. 

The KFPs extraction can be the first step for AIS big data analysis and research, and the subsequent research can be directly carried out based on these KFPs. Hence, studying KFP extraction algorithms for AIS trajectory data has become an urgent requirement. Identifying representative points from the massive AIS trajectory and extracting KFPs is a pre-processing method for AIS data. Some AIS data points possess low value, and the original trajectory of the ship is not affected after their removal. The expression of the other part is crucial for the description of the trajectory features. Once the ship trajectory is removed, huge distortions can occur. The point where the value of such a trajectory is higher is defined as a KFP in this paper.

Most feature point extraction algorithms in this field focus on methods such as image, video, human face, point cloud, graphic boundary, and moving-object trajectory KFP extraction. Most of these methods are based on visual feature extraction. These studies rely on image information to study the ship trajectory, which can be extremely difficult and expensive because of the distance between a ship and land. It is difficult to clearly capture image data for using shore-based camera equipment. Such camera equipment is not mandatory on ships because it is highly costly and impractical to install. Notably, there are relatively few studies on feature extraction for ship navigational behavior that combine spatiotemporal characteristics.

Conventional KFP extraction algorithms can be classified into the following three types:

(a) Image, human face, and facial feature extraction, which are commonly accomplished using neural networks [12], propagation filters [13], support vector machine (SVM) [14], etc.;
(b) Point cloud feature extraction, which is commonly accomplished using Gaussian normal clustering [15], multi-scale tensor voting [16], etc.;
(c) Line segment, moving object trajectory, and graphic boundary feature point extraction, which are commonly accomplished using a Kalman filter [17], trip frequency and accumulated distance [18], compression algorithms, etc.

Compression algorithms provide an extremely effective, simple, and fast processing method for the extraction of the KFPs of moving objects. Whether using a compression algorithm or feature extraction algorithm, the approach is essentially a system for evaluating the value of trajectory expression. The compression algorithm accomplishes compression by removing the points with low trajectory values in data. Feature extraction maintains the points with high trajectory values in data as the representative points of a trajectory, which are also the KFPs. The research related to compression algorithms includes the work by Jiang et al. [19], who obtained the trajectory characteristics of vehicles in images. Based on an algorithm for polygonal linear scanning calibration, considering the influence of the distortion of charge-coupled device (CCD) cameras, and introducing the center deviation factor, multi-region scanning calibration considering a center offset (MRSCCO) algorithm was proposed to transform the trajectory features of images into actual motion features. Wang et al. [20] proposed a novel fusion-based ship detection method from polarimetric synthetic aperture radar (Pol-SAR) images to extract the trajectory features of ships from polarimetric synthetic aperture radar images. Tu et al. [21] used the Hilbert transform (HT) and wavelet transform modulus maximum (WTMM) on the terahertz pulsed imaging (TPI) of ship coatings to extract structural features. Mi and Huang [22] constructed a new time series analysis model for network traffic time series data based on quantitative recursive feature extraction, in which the percentage of frequency characteristic points among the total points in a plane was calculated in a recursive figure plane, and the features of network traffic time-frequency entropy were extracted. Stefani et al. [23] proposed a new method for processing digitized radar images to extract ship features. Wang and Zeng [24] used a close-up camera to collect the images of moving vehicles, and the motion trajectories of vehicle feature points were obtained from successive images and converted into Cartesian coordinates to extract vehicle trajectories. Sang et al. [8] proposed three trajectory characteristics (line, curve, and arc), and five trajectory steps (line, curve, arc, curve, and line) during the turning section. Gerben et al. [11] proposed a piecewise linear segmentation (PLS) algorithm that could compress a trajectory into linear segments by recursively keeping the points that have a maximum error higher than a fixed threshold. Fu et al. [25] invoked reference points as well as PLS and applied a compression method based on the routing and movement features. Sheng et al [26] extracted 17 types of trajectory features based on the basic movement patterns and the sub-trajectory segments and divided the ship trajectory into three basic movement patterns (anchored-off pattern, turning pattern, and straight-sailing pattern). Many researchers (Zhu et al [10], Li et al [27], Zhang et al [9,28,29,30], Zhao et al [31,32], Mou et al [33], Ren et al [34]) have used the Douglas–Peucker (DP) algorithm for the pre-processing of AIS big data. However, this algorithm has high time complexity and it is time-consuming to run. 

Furthermore, its operational complexity is extremely high, and thus, it can easily cause computer memory overflow, which is particularly unsuitable for large quantities of AIS data. Zhu and Sun [35] proposed a minimum bounding rectangle (MBR) GPS trajectory data compression algorithm. However, it was difficult to determine the number of discrete points included in a rectangle using this algorithm. Furthermore, the rectangle direction was not considered, and the point selection strategy became highly complex when the rectangular area was extremely large or extremely small. Dutta et al. [36] proposed a reservoir-sampling data compression algorithm. It does not consider the time sequence, spatial sequence, or the importance of other factors. As a result, it may generate errors. Yang et al. [37] proposed a finger vein region of interest (ROI) extraction method that is robust against finger displacement and rotation. A sliding window was used to detect phalangeal joints and to ascertain the height of the ROI for a corrected image.

Most algorithms use the Euclidean vertical distance for the selection criterion of trajectory KFPs, in which only the spatial position characteristics of a ship are considered. The time characteristics of the ship are only used as an index for sorting [38], and the spatiotemporal characteristics of ship AIS data are considered in even fewer cases. In recent years, the research on the mining of ship AIS data has slightly matured. However, the combination of spatiotemporal characteristics in ship trajectory data is rare. Otsuka et al. [39] considered the spatial arrangement of dominant contours, the uniformity of velocity components, and trajectory run length to extract the KFPs from a motion trajectory. Cao et al. [40] proposed the adoption of line simplification, which is a technique that is typically applied in computer graphics, to approximate and reduce spatiotemporal AIS data. Satoh et al. [41] proposed a method for recognizing the 3D shape of a moving object using active cameras and proposed a binocular motion tracking system by reconstructing the 3D shape of various moving objects. Sandu et al. [42] proposed an extended data model and a network partitioning algorithm using long paths to increase the compression rates for the same error bounds. In this study, we integrated these proposals with a state-of-the-art compression algorithm to obtain a new technique for compressing network trajectory data using deterministic error bounds. 

Currently, analysis of spatiotemporal information is on the frontier of the research in the field of data mining. Ship AIS data are multi-sensor [43] data that include the position, time, ship navigation status, heading, and speed information of ships. Therefore, these datasets contain recorded ship static, dynamic, and navigational information for a variety of activities and provide an effective method of accurately identifying ship motion characteristics. This work combines the ship’s navigational behavior characteristics with the spatiotemporal characteristics of ship AIS data to improve the sliding window compression algorithm and extract a ship’s behavioral KFPs, thereby facilitating effective AIS trajectory data mining and providing a foundation for subsequent research on ship handling behavior and intelligent ship collision avoidance.

## 2. Feature Extraction Method 

The development of big data analysis of AIS data has become popular owing to the completion of AIS infrastructure and the continued development of AIS technologies. Eventually, the order of magnitude of the data will be too large for computers to handle. This is the major disadvantage of offline feature extraction algorithms such as the DP algorithm, as they can only extract feature data that have been analyzed or stored. Furthermore, they depend on the end point to judge all points. 

**Figure 2:**
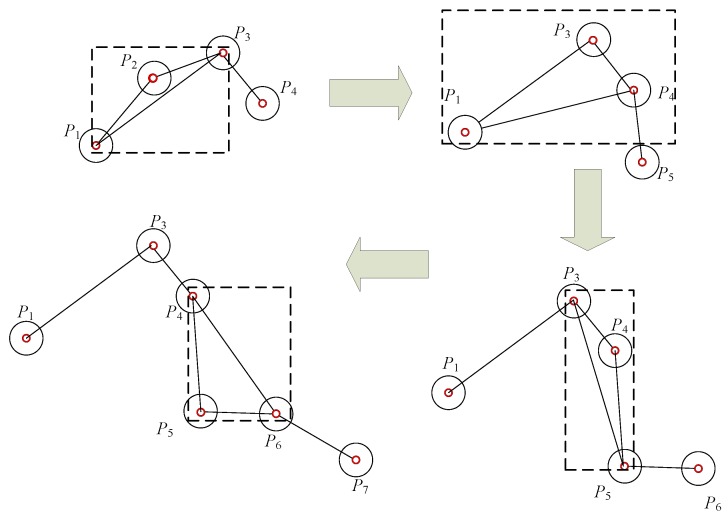
Sliding window rectangle in automatic identification system (AIS) trajectory extraction.

The sliding window has been widely used in deep learning in both convolutional and pooling layers. The receptive field of the sliding window in convolutional neural networks (CNNs) is a 3D box that achieves the goal of processing an entire large image by sliding the window. The receptive field of the sliding window in AIS trajectory extraction is a rectangle that always contains only three points and only judges whether the second point is retained as a feature point in the track at a certain time, as shown in Figure 2 [44,45]. Therefore, in this study, we propose an online sliding window feature extraction algorithm. In this study, we combined the principle of the compression algorithm, the ship navigation angle deviation, position deviation, and AIS spatiotemporal characteristics to improve the performance of the online sliding window KFP extraction algorithm. 

### 2.1. Improved Sliding Window Algorithm Based on Spatiotemporal Characteristics

**Figure 3:**
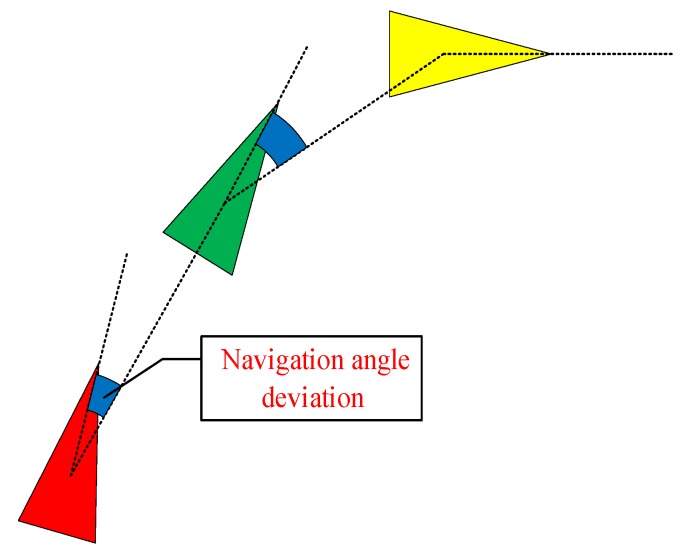
Navigation angle deviation.

The ship navigation angle deviation is shown in Figure 3. According to the Markov chain [46], its value in future states is only related to the current state. Regardless of the previous state, the deviation in angle of the next point is determined according to the state of the previous point. Further enlargement studies show that the ship is no longer simplified as a point, considering the ship direction and heading in the plane. Most algorithms always use the Euclidean vertical distance for the selection criterion of trajectory KFPs, in which only the spatial position data of a ship are considered. The time information in AIS data is only used as an index for sorting, and the spatiotemporal characteristics of ship AIS data are considered in even fewer cases [47].

**Figure 4:**
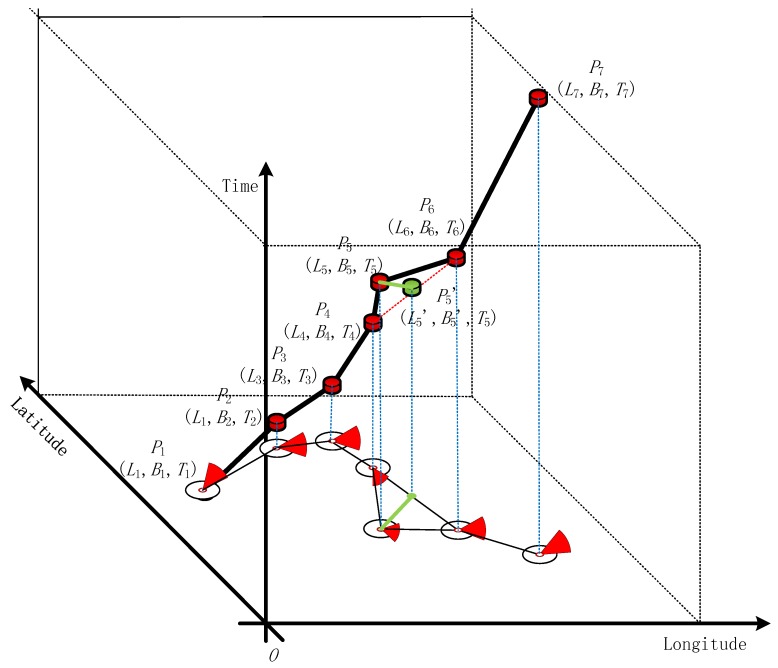
3D trajectory and its projected path in the 2D plane.

**Figure 5:**
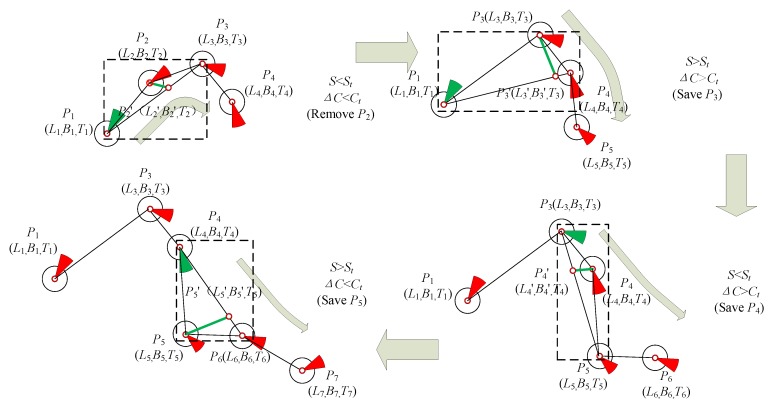
Schematic of the proposed improved sliding window algorithm.

**Figure 6:**
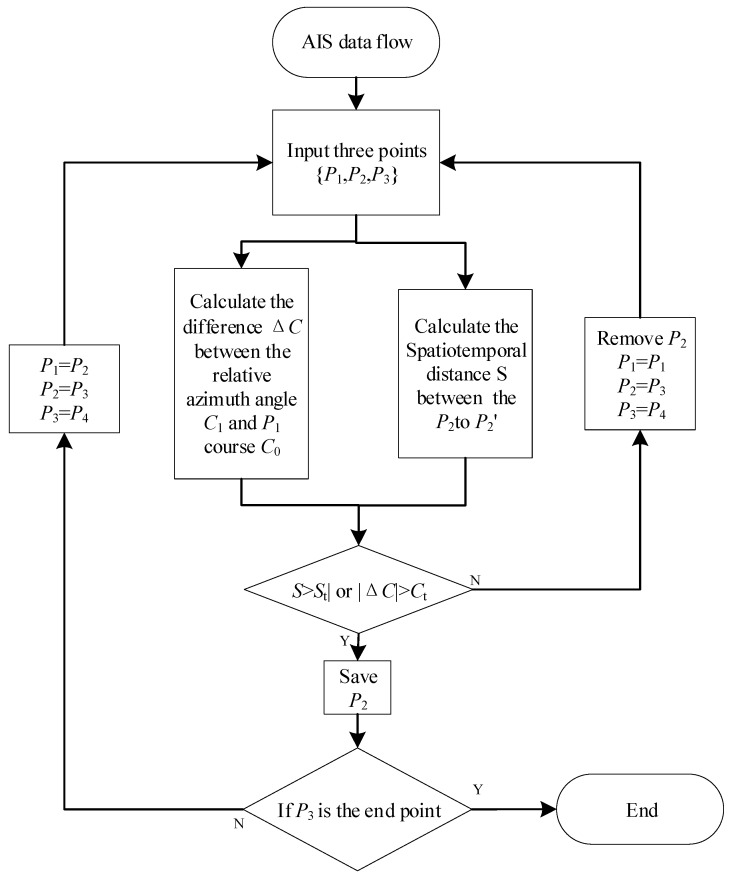
Flow diagram of the proposed improved sliding window algorithm.

The feature extraction process is briefly described as follows: First, points *P*_1_ (*L*_1_, *B*_1_, *T*_1_), *P*_2_ (*L*_2_, *B*_2_, *T*_2_), and *P*_3_ (*L*_3_, *B*_3_, *T*_3_) are located in the initial window. Using time *T*_2_ of point *P*_2_ (*L*_2_, *B*_2_, *T*_2_) as an index, the difference *S* between *P*_1_ (*L*_1_, *B*_1_, *T*_1_) and *P*_3_ (*L*_3_, *B*_3_, *T*_3_) can be calculated as *P*_2_*’* (*L*_2_’, *B*_2_’, *T*_2_). If spatiotemporal distance *S* is less than the threshold, then the relative azimuth angle from *P*_1_ to *P*_2_ is calculated and named *C*_1_. The *P*_1_ course is referred to as *C*_0_. The difference ΔC between *C*_1_ and *C*_0_ must be less than the calculated angle threshold. If this condition is satisfied, *P*_2_ can be deleted. In this manner, feature extraction is accomplished. The improved sliding window algorithm is illustrated in Figure 4, Figure 5 and Figure 6. 

These parameters are calculated as follows,

(1) $$\Delta i=T_{i}-T_{s},$$

(2) $$\Delta e=T_{e}-T_{s},$$

(3) $$B_{i}^{\prime }=B_{s}+\frac{\Delta i}{\Delta e}(B_{e}-B_{s}),$$

(4) $$L_{i}^{\prime }=L_{s}+\frac{\Delta i}{\Delta e}(L_{e}-L_{s}),$$

where B is AIS latitude, L is AIS longitude, T is AIS time, Δi is the time elapsed from the initial point to the check point, and Δe is the time elapsed from the initial point to the end point.

Then, the spatiotemporal distance from *P*_2_ to *P*_2_’ is calculated. The rhumb line anti-inference method is applied for calculating the orientation of two ships. Given two-point coordinates, ($L_{1},B_{1}$) and ($L_{2},B_{2}$), the solution equation is as follows,

(5) $$\left\{ \begin{array}{l} \Delta B=B_{2}-B_{1}\\ \Delta L=L_{2}-L_{1} \end{array}\right.,$$

(6) $$s_{B}=a(1-e^{2}){\int_{0}^{B}\left(1-e^{2}\sin^{2}B\right)}^{-3/2}\Delta B,$$

where ΔB is the latitude difference in the geodetic coordinate system, ΔL is the longitude difference in the geodetic coordinate system, a is the major radius of the ellipsoid, e is the eccentricity, and $s_{B}$ is the arc from the evaluated point to the equator. 

If ΔL>π, ΔL=ΔL−2π is applied until ΔL≤π; if ΔL<−π, ΔL=ΔL+2π is applied until ΔL≥−π in

(7) {q=ln[tan(π4+B2)(1−esinB1+esinB)e2]ΔX=adLΔY=a(q2−q1)C=arctan2(ΔX,ΔY),

(8) $$S=\left\{ \begin{array}{l} \frac{\left(s_{B_{2}}-s_{B_{1}}\right)}{\cos A_{1}}\;(\Delta B\neq 0)\\ r_{1}|\Delta L|\;(\Delta B=0) \end{array}\right.,$$

(9) $$r_{1}=\frac{a\cos B_{1}}{\sqrt{1-e^{2}\sin^{2}B_{1}}},$$

where q is the isometric latitude, $r_{1}$ is the latitude radius at latitude *B*_1_, ΔX is the horizontal coordinate difference in the Mercator coordinate system, *A*_1_ is starting point azimuth, ΔY is the vertical coordinate difference in the Mercator coordinate system, C is the angle of the straight line between two ship points, and S is the length of the straight line between two ship points.

### 2.2. Feature Extraction Effect Verification 

**Figure 7:**
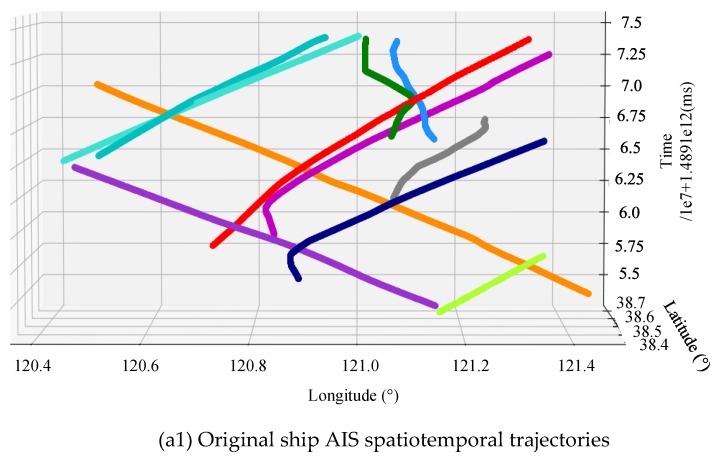

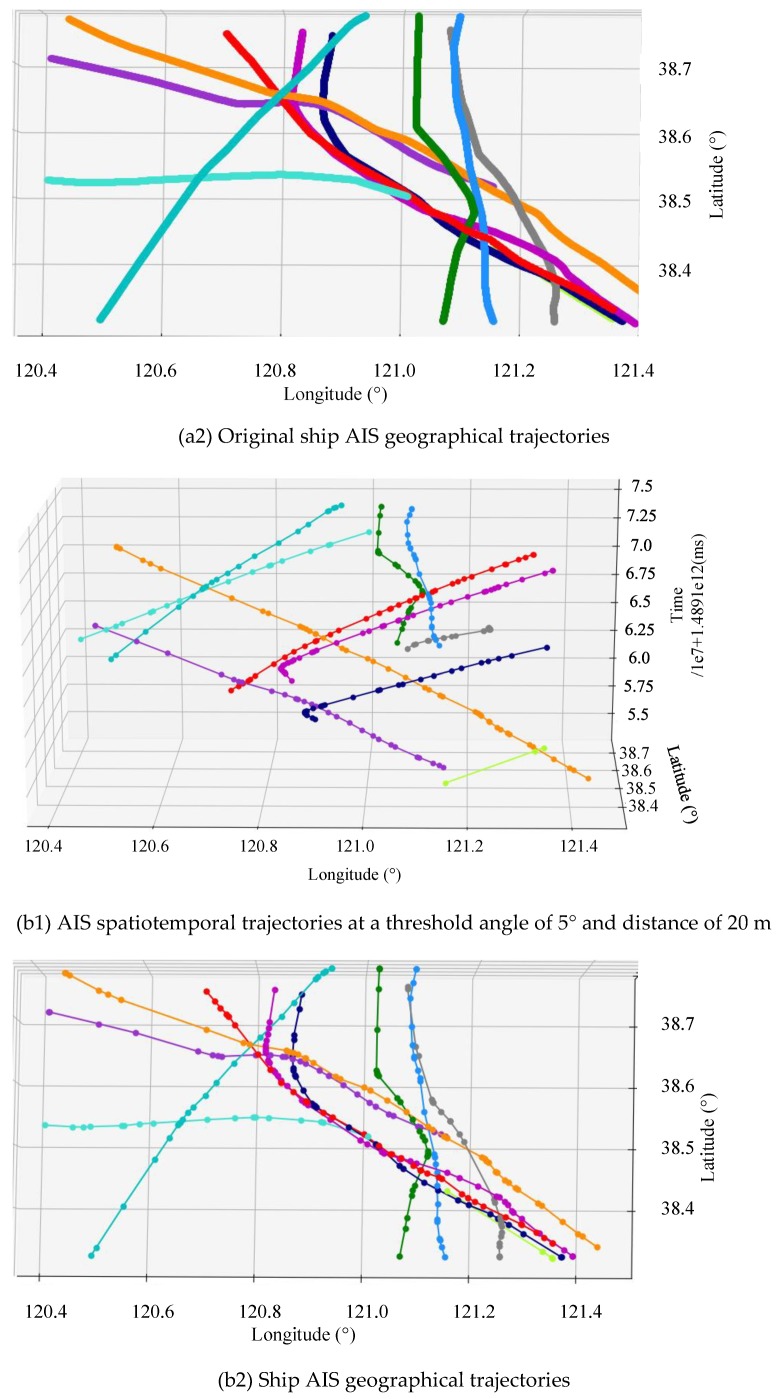

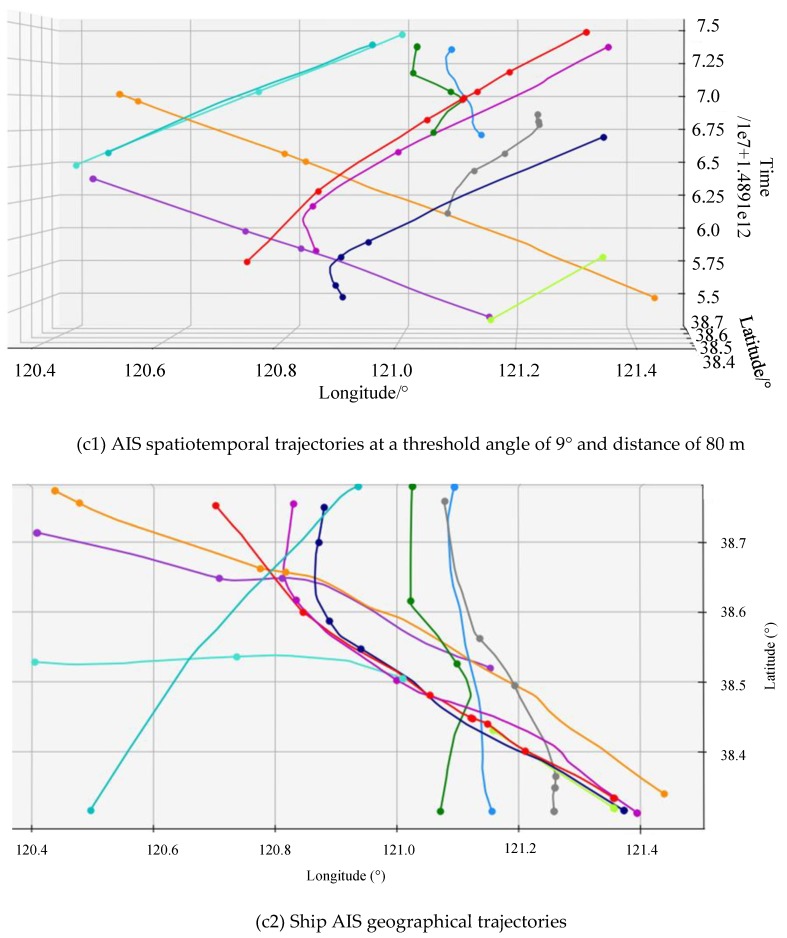
Ship trajectory feature extraction using different thresholds.

We obtained and analyzed a ship’s AIS trajectory data to demonstrate the effect of different thresholds on KFP extraction. Then, different distance and angle thresholds were set to generate the different KFP extraction results shown in Figure 7.

As seen in Figure 7, the redundant information rejection ratio increases with the sliding window threshold. The amount of information decreases with the number of extracted points. When the thresholds reach a certain level, it is possible that valid KFPs could be removed as if they were redundant points. Thus, when applying the proposed KFP extraction algorithm, a reasonable threshold must be selected to appropriately extract KFPs. 

## 3. Feature Extraction Threshold Determination

Ship handling behavior pattern recognition and navigation experience learning influence KFPs. In this process, redundant data can be reduced to achieve feature extraction. However, deleting trajectory data inevitably leads to distortion. In the region of a high redundant information rejection ratio, the rejection ratio is positively related to the distortion. The higher the rejection ratio, the higher the distortion. Thus, a high rejection ratio cannot be blindly considered. An expected threshold range must be determined based on the trade-off between the rejection ratio of redundant information and acceptable distortion. The rejection ratio and the distortion are a pair of interdependent relational variables. Our study aims to reduce the distortion to an acceptable range. However, it is impossible to directly quantify the distortion. Therefore, in this study, we calculate the redundant information rejection ratio to quantify the distortion indirectly.

**Figure 8:**
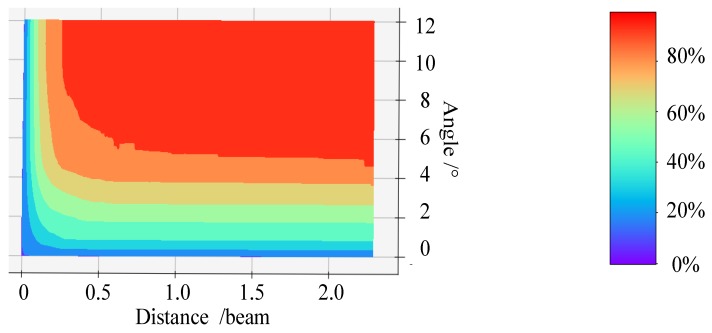
Diagram of the redundant information rejection ratio.

Different feature extraction thresholds produce different feature extraction results. Thus, the selection of a suitable feature extraction threshold ensures that AIS trajectory data are extracted effectively and appropriate KFPs are retained. To determine the appropriate feature extraction threshold, experimental feature extraction was performed for the Cheng Shantou channel in March 2015 using AIS trajectory data. The data contain information on the AIS trajectory of 332 ships, consisting of 926,497 points. It is impossible for ships of different sizes to share the same distance threshold; therefore, it must be properly adjusted by the size of the ship. In order to facilitate its quantification and improve versatility, a unitization process was conducted on the distance threshold. Referring to the ship-domain model proposed by Fujii, the ellipse-shaped ship domain is determined to be 8 times the length of the ship in its major axis, and 3.2 times the length of the ship in its minor axis [48]. In this study, the distance threshold is set as a parameter to describe the left and right direction distance offsets for the ship. Compared with the length of the ship, the ship beam is also the distance parameter in the left and right direction. Therefore, we used the beam of ship as the parameter for the unitization of the distance threshold. Then, the distance threshold was set between 0 to 2.5 times the beam of the ship, and the ship navigation angle deviation threshold was set between 0° to 12°. The corresponding KFPs were obtained using the proposed AIS trajectory feature extraction method. The redundant information rejection ratio diagram is shown in Figure 8.

**Figure 9:**
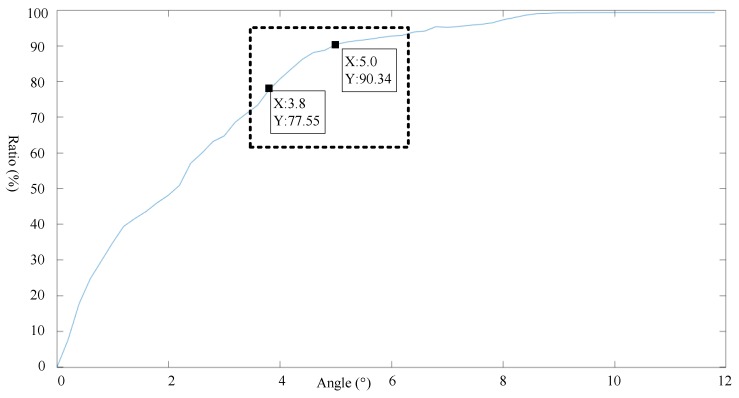
Relationship between the angle threshold and feature extraction ratio for a distance threshold of 59 m.

**Figure 10:**
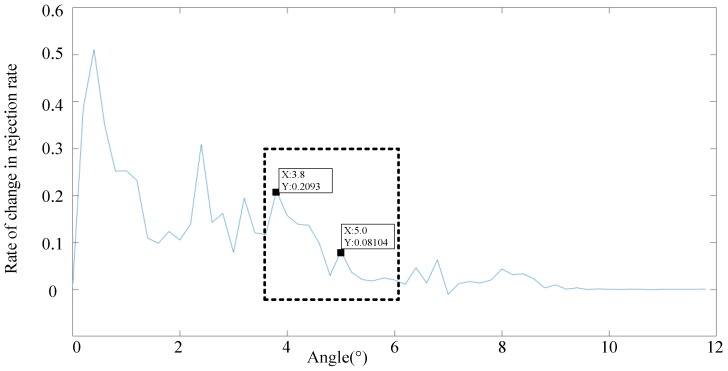
Relationship between the angle threshold and rate of change in rejection rate for a distance threshold 1.7 times the beam.

According to Figure 8, the influence of the angle threshold is clearly stronger than that of the distance threshold. As a result, we set the angle threshold first, as shown in Figure 9 and Figure 10, where a distance threshold that is 1.7 times the beam is selected.

**Figure 11:**
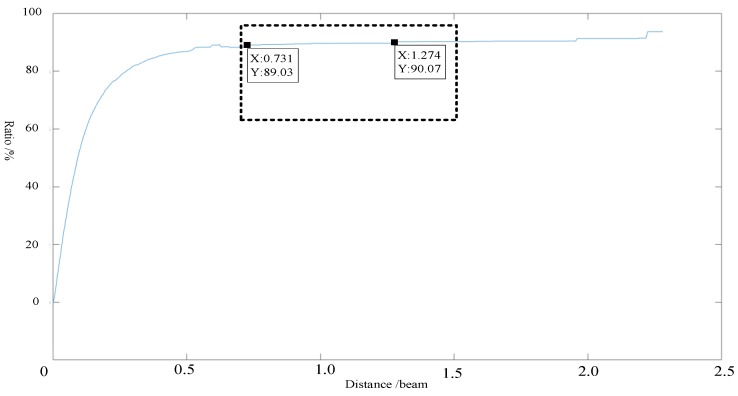
Relationship between the distance threshold and the redundant information rejection ratio for an angle of 5°.

**Figure 12:**
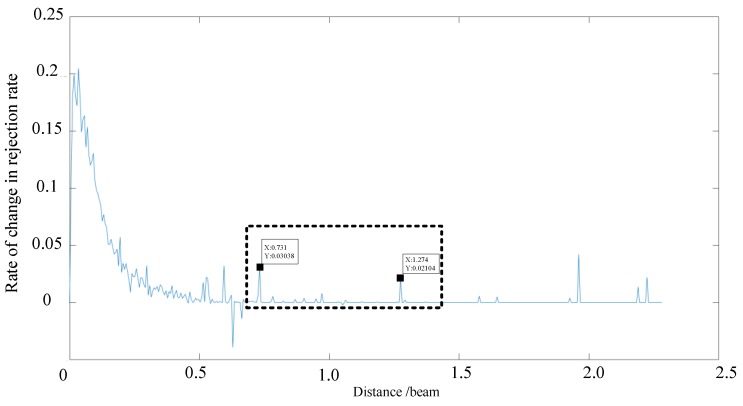
Relationship between the distance threshold and rate of change in rejection rate for an angle of 5°.

Similarly, the threshold data obtained using an angle threshold of 5° were analyzed to determine the influence of the distance threshold and the relationship between the redundant information rejection ratio and distance threshold. As shown in Figure 11 and Figure 12, when a distance threshold of 5° m is selected, the angle threshold is determined as (3.8–5.0°).

We determined the recommended thresholds for feature extraction (0.731–1.274, 3.8–5.0°) to assist in the selection of the distance and angle thresholds when applying the proposed algorithm. These thresholds must be re-established for different applications. In order to achieve versatility for use in other ships, this threshold has a certain relationship with the beam of ship. A relative value is obtained as the recommended initial threshold compared with the beam of ship. The recommended feature extraction thresholds were converted to dimensionless indices. The distance threshold was (0.731–1.274) times the beam of the ship. These thresholds are suitable considering limited storage space because they provide a good feature extraction ratio. 

## 4. Results and Discussion of KFP Extraction Analysis

After selecting the appropriate threshold, the sliding-window algorithm was applied to extract ship behavior KFPs from AIS trajectories. The superiority of the DP algorithm has already been demonstrated for offline applications. In this section, we compare the computational efficiencies of the DP algorithm and improved sliding window algorithm. 

The concept of the DP algorithm is to add KFPs to achieve feature extraction. However, the DP algorithm is likely to miss critical KFPs because extracted data exhibit significant distortion when the redundant information rejection ratio is extremely high. The concept of the proposed online sliding window feature extraction algorithm is to remove corresponding redundant points under feature extraction. The AIS tracking data for a ship were selected to demonstrate Tianjin Port in December 2017 using AIS trajectory data. The data containing information on the AIS trajectory of 1067 ships, consisting of 899,854 points, were extracted using the DP algorithm and improved sliding window algorithm. The distance thresholds were set as in the range of 0.25–3 times the ship beam with increments of 0.25, and an angle threshold of 4.5° was used in the sliding window algorithm for feature extraction. Then, the two algorithms were compared in terms of their running time to evaluate their computational efficiency, as feature extraction efficiency can be quantified by algorithm running time. The results of the analyses are presented in Table 1. 

**Figure 13:**
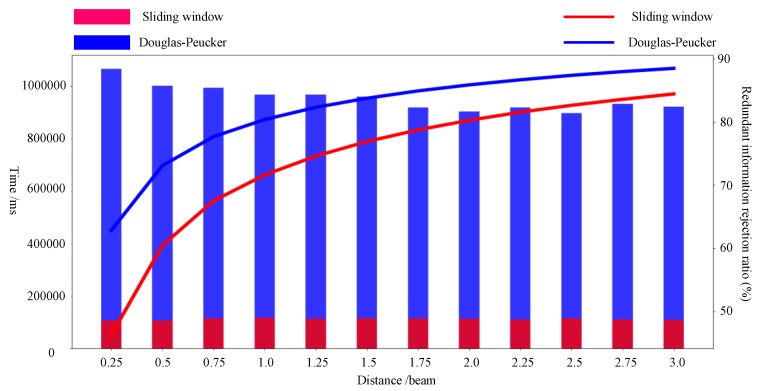
KFP extraction efficiency of the two evaluated algorithms comparison chart.

**Figure 14:**
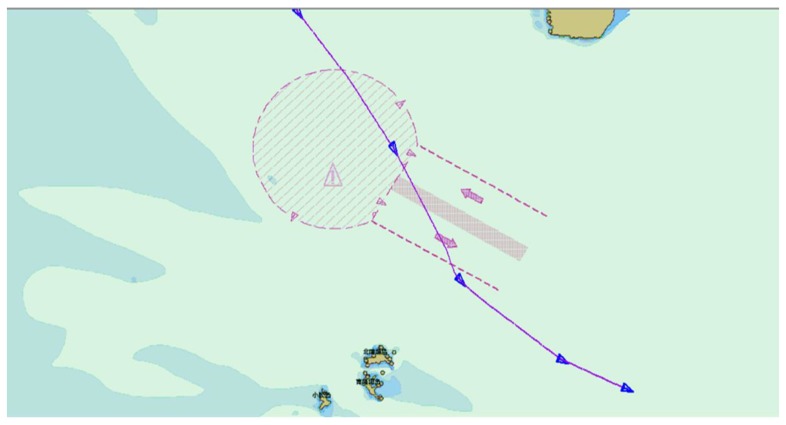
AIS trajectory KFP extracted by Douglas–Peucker (DP).

**Figure 15:**
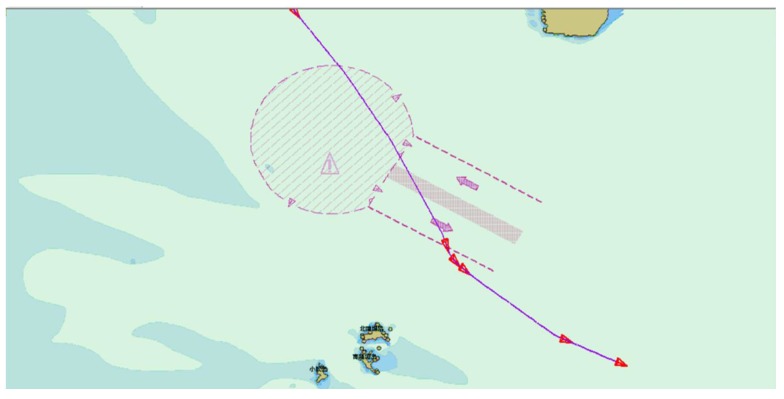
AIS trajectory KFPs extracted by sliding window.

As seen in Table 1 and Figure 13, even though the redundant information rejection ratio of the DP algorithm is higher than that of the improved sliding window algorithm, in the region of high rejection ratio, the rejection ratio is positively related to the distortion. The higher the rejection ratio, the higher the distortion. The DP algorithm pursues the redundant information rejection ratio, which causes a certain degree of distortion. In Figure 14 and Figure 15, the purple lines represent the original AIS trajectory, the blue ship represents the KFPs extracted by the DP algorithm, and the red ship represents the KFPs extracted by the sliding window algorithm. The results extracted by the sliding window algorithm indicate that it can preserve more ship turning details than the DP algorithm, which preserves the rough shape of the trajectory; the remaining points can be displayed, and the extracted KFPs cannot be used in subsequent trajectory behavior studies. Therefore, the comparison shows that the sliding window not only performs better when extracting trajectory feature points, but also has a higher extraction efficiency.

Meanwhile, in terms of time, the sliding window algorithm requires an average of 112,216 ms, while the DP algorithm requires on average 954,279.5833 ms. The results indicate that the DP algorithm performs slightly better KFP extraction. However, the improved sliding window algorithm performs KFP extraction considerably faster, potentially compensating for the decrease in KFP extraction performance. 

## 5. Conclusions

The transmission, display, learning, and storage of AIS information require considerable time, computing power, and storage space. In this paper, an improved sliding window algorithm that combines the spatiotemporal characteristics of ship AIS trajectory data was developed and tested to extract KFPs. The proposed algorithm significantly improves the computational efficiency of AIS trajectory data processing and provides a theoretical basis for future research on ship AIS trajectory data processing and ship behavior feature pattern recognition. The recommended threshold of the ship navigation angle deviation was found to be (3.8–5.0°) through data fitting using a distance threshold that was (0.73–1.27) times the beam of the ship. Given the recommended KFP extraction threshold, the users of the proposed algorithm can select an appropriate threshold, which is biased toward KFP extraction accuracy and efficiency, according to their KFP extraction requirements. The recommended threshold provided in this paper can also be used as the initial threshold for KFP extraction processing, and additional adjustments can be made as required for a particular ship. The results of this study demonstrate that the proposed improved sliding window algorithm that combines spatiotemporal characteristics can be applied to rapidly and easily extract KFPs from AIS trajectory data. The KFPs in the ship trajectory data contain the framework of the overall trajectory. The subsequent research on AIS big data can be directly carried out on the basis of KFPs. KFPs can be used as the nodes of the trajectory. The ship trajectory can be cut off at KFPs to generate a sub-trajectory. This ability is extremely significant for the analyses of traffic flow and the navigational behavior of ships.

## Figures and Tables

**Table 1:** KFP extraction efficiency of the two evaluated algorithms.

	DP Algorithm		Improved Sliding Window Algorithm (For Angle Threshold of 4.5°)	
Distance Threshold (/beam)	Time (ms)	Redundant Information Rejection Ratio (%)	Time (ms)	Redundant Information Rejection Ratio (%)
0.25	1,065,880	62.8326	106,738	46.2627
0.5	1,001,909	73.1039	107,405	60.4662
0.75	993,541	77.6836	115,015	67.5149
1	968,285	80.4005	117,772	71.6335
1.25	967,549	82.3278	114,028	74.6086
1.5	960,610	83.7742	114,211	76.9160
1.75	919,216	84.9347	114,143	78.7610
2	903,670	85.8901	113,539	80.2842
2.25	918,580	86.6922	110,629	81.5669
2.5	897,767	87.3986	114,095	82.6549
2.75	932,628	87.9897	110,449	83.5983
3	921,720	88.5085	108,568	84.4530
